# Commentary: Melanopsin Regulates Both Sleep-Promoting and Arousal-Promoting Responses to Light

**DOI:** 10.3389/fncir.2016.00094

**Published:** 2016-11-16

**Authors:** Christian Cajochen, Sarah L. Chellappa

**Affiliations:** ^1^Centre for Chronobiology, Transfaculty Research Platform Molecular and Cognitive Neurosciences, Psychiatric Hospital of the University of Basel, University of BaselBasel, Switzerland; ^2^Medical Chronobiology Program, Division of Sleep and Circadian Disorders, Brigham and Women's Hospital and Division of Sleep Medicine, Harvard Medical SchoolBoston, MA, USA

**Keywords:** light, melanopsin, sleep, wake, neuronal networks

## A role for melanopsin in sleep and wake neural circuits

**Figure 1:**
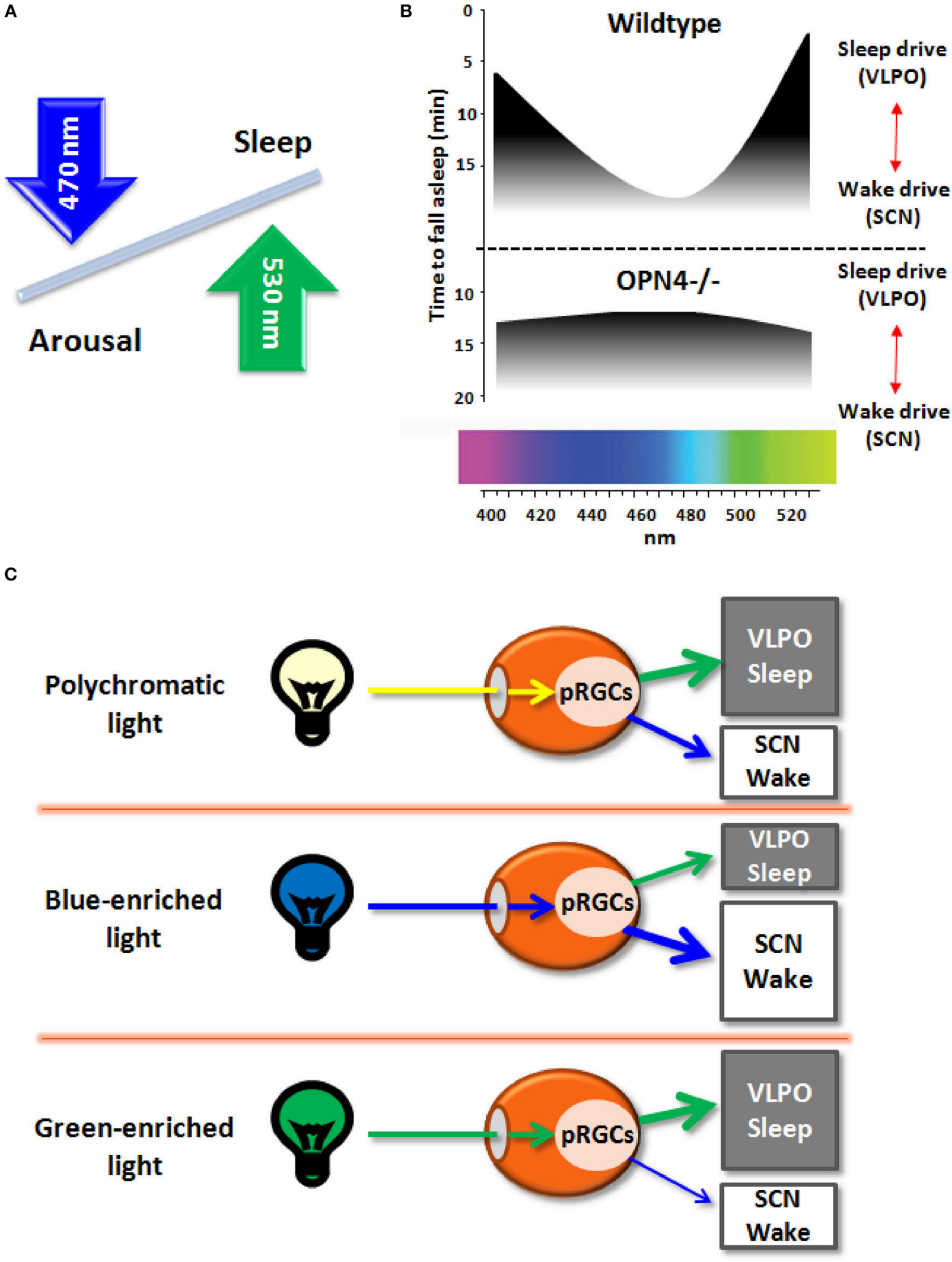
**(A)** Schematic diagram of the effects on light wavelengths (blue and green light) on sleep and arousal states. **(B)** Melanopsin-deficient mice (Opn4^−/−^), as compared to wildtype mice, exhibit blunted responses to non-image forming effects of light, with increased sleep in response to blue light (peak sensitivity around 480 nm) and less sleep induction in response to green light (peak sensitivity around 530 nm). **(C)** Broadband polychromatic white light potentially impacts on both sleep and wake states, while blue light (short wavelength) actively fosters an enhanced wake state, and green light (longer wavelength) may elicit sleep-promoting effects.

The recent discovery of melanopsin (OPN4), a photopigment maximally sensitive to the blue spectrum of light (peak sensitivity ~480 nm) and expressed in a subset of photosenstive retinal ganglional cells (pRGCs) within the eye (Berson et al., 2002; Schmidt et al., 2011), paved the ground to better understand the non-image forming (NIF) effects of light. Besides photic resetting of the circadian clock, light striking the retina between dusk and dawn impacts on neurons regulating sleep-wake cycles, by inhibiting sleep-promoting neurons and activating arousal-promoting orexin neurons in the hypothalamus (Saper et al., 2005). While the role for melanopsin in mediating light effects on sleep/arousal is known (Figure 1A; Tsai et al., 2009), it remains to be established if the spectral composition of light (i.e., wavelengths) modulates these effects.

Very recently, Pilorz et al. (2016) pursued this question by investigating the effects of different light wavelengths on acute sleep and arousal induction in rodents and how melanopsin mediates these effects. Using a multimodal approach, ranging from behavior to hormonal and gene expression responses, a complex role for melanopsin in sleep-wake regulation was yielded. By exploiting the melanopsin knockout mouse (OPN4^−/−^) as compared to wildtypes, the authors could convincingly show that both opposing effects of light rely on melanopsin pRCGs through potentially different pathways: blue-light active retinal projections to the suprachiasmatic nuclei (SCN; central circadian pacemaker) and green-light active projections to the ventrolateral preoptic area (VLPO; major sleep promoting area) that mediate arousal and sleep induction, respectively (Figure 1B). Furthermore, glucocorticoid release by light during the night, a physiological proxy for arousal-promoting light effects, was mediated by SCN-projecting melanopsin pRCGs. The data suggest that blue light promotes a state of arousal that counteracts the sleep-promoting effects of longer wavelength light (Figure 1C). Accordingly, the ratio between the blue and green portion of the animal's light input defines the somnogenic/alerting response to a given light scenario (Figures 1A–C). The arousing effects of blue light are consistent with human data on alertness, melatonin suppression and sleep-wake regulation (Cajochen et al., 2005; Chellappa et al., 2011, 2013). However, it remains uncertain if the response to green light may depend on nocturnality/diurnality. As no direct comparison of blue and green light exposure has been conducted simultaneously in nocturnal and diurnal species, the opposing effects of blue and green light on vigilance states remain to be fully established. Importantly, these results confirm the role of melanopsin in the drive for arousal rather than sleep in both nocturnal animals and humans, since melanopsin loss lead to attenuated arousal response and more rapid sleep induction in OPN4^−/−^ mice. The response to green light comes as a surprise since its sleep-promoting effects have not been reported before in humans and nocturnal rodents, except for higher human EEG activity in the delta and sleep spindle range in occipital brain regions after evening exposure to monochromatic light at 550 nm (green light; Münch et al., 2006).

The neuroanatomical circuits mediating blue and green-light effects on sleep and arousal, respectively, may temptingly account for the specificity of these effects (Morin, 2015). A critical input pathway for photic somnolence/arousal involves a relay in one or more of the hypothalamic nuclei to the sleep regulatory system, with the SCN being the prime candidate. Orexin (hypocretin) cells in the lateral hypothalamus (LH) are direct SCN targets and major constituents of the sleep/arousal system (Saper et al., 2010). Interestingly, orexin release has both direct and indirect inhibitory effects on SCN activity, and may underlie how sleep-/arousal-related stimuli adjust circadian rhythm function (Belle et al., 2014). If pRGC stimulation through light at shorter wavelength (blue) relays to the SCN and thereof to orexinergic cells in the LH, one could assume a (simplified) pathway for arousal-mediating effects. Conversely, the sleep-promoting green-light effects are more complex to reconcile. One hypothesis is that their effects could be “indirect.” Previous studies indicate a multisynaptic route involving the LH orexinergic system and the VLPO (Yoshida et al., 2006; Saper et al., 2010). As green light is not known to actively promote a putative arousal-pathway, LH orexinergic neuronal activity might be decreased by exposure to this light setting. This would allow for increased activity within the VLPO neurons, thus ensuring a sleep-promoting signal. However, the specific pathways for sleep/arousal promoting effects by different light wavelengths remain elusive. Despite these gaps of knowledge, one can affirm that, besides melanopsin's role in mediating NIF responses on sleep, rods and cones most likely contribute to these NIF effects. Yet, the specific role of melanopsin pRCGs, rods and cones innervating brain regions beyond the SCN, like the subparaventricular zone (SPZ), which directly projects to the dorsomedial hypothalamus (DMH) that regulates the VLPO and LH (Muindi et al., 2014), warrants detailed investigation. A potential limitation to Pilorz and colleagues is the absence of a dose-response relationship for different light wavelengths, as the “green effect” might reflect lower “melanopic strength” (illuminance). Thus by reducing the melanopic strength of a blue light stimulus, it could act similarly as green light. Although the proposed model of a clear dichotomy between blue light-SCN-wake and green light-VLPO-sleep axis may be oversimplified, it offers a framework to be tested in further animal but also clinical human sleep research settings. In other words, if different light wavelengths are proven to enhance alertness and sleep in humans, light will have a great potential therapeutic perspective for patients suffering from sleep disorders, such as insomnia, hypersomnia, excessive daytime sleepiness, and circadian sleep-wake disorders. Human biology has a direct and ancestral history with natural sunlight. Yet, contemporary lifestyle, which is heavily dependent on artificial light sources, including computers, TV, self-illuminated personal electronic devices, indoor, and street lighting, challenges this ancestral relationship (Cajochen et al., 2011; Chang et al., 2015). The exciting findings by Pilorz and colleagues provide a substantial basis to better understand NIF effects of light to adapt our illuminated surroundings such that we can positively influence our sleep and circadian physiology.

## Author contributions

CC wrote the general commentary and created the graphic illustration. SC wrote the general commentary.

### Conflict of interest statement

The authors declare that the research was conducted in the absence of any commercial or financial relationships that could be construed as a potential conflict of interest.
